# Suilysin Stimulates the Release of Heparin Binding Protein from Neutrophils and Increases Vascular Permeability in Mice

**DOI:** 10.3389/fmicb.2016.01338

**Published:** 2016-08-26

**Authors:** Shaolong Chen, Wenlong Xie, Kai Wu, Ping Li, Zhiqiang Ren, Lin Li, Yuan Yuan, Chunmao Zhang, Yuling Zheng, Qingyu Lv, Hua Jiang, Yongqiang Jiang

**Affiliations:** State Key Laboratory of Pathogen and Biosecurity, Beijing Institute of Microbiology and EpidemiologyBeijing, China

**Keywords:** heparin binding protein, *Streptococcus suis*-associated streptococcal toxic shock syndrome, suilysin, vascular permeability

## Abstract

Most of the deaths that occurred during two large outbreaks of *Streptococcus suis* infections in 1998 and 2005 in China were caused by streptococcal toxic shock syndrome (STSS), which is characterized by increased vascular permeability. Heparin-binding protein (HBP) is thought to mediate the vascular leakage. The purpose of this study was to investigate the detailed mechanism underlying the release of HBP and the vascular leakage induced by *S. suis*. Significantly higher serum levels of HBP were detected in Chinese patients with STSS than in patients with meningitis or healthy controls. Suilysin (SLY) is an exotoxin secreted by the highly virulent strain 05ZYH33, and it stimulated the release of HBP from the polymorphonuclear neutrophils and mediated vascular leakage in mice. The release of HBP induced by SLY was caused by a calcium influx-dependent degranulation. Analyses using a pharmacological approach revealed that the release of HBP induced by SLY was related to Toll-like receptor 4, p38 mitogen-activated protein kinase, and the 1-phosphatidylinositol 3-kinase pathway. It was also dependent on a G protein-coupled seven-membrane spanning receptor. The results of this study provide new insights into the vascular leakage in STSS associated with non-Group A streptococci, which could lead to the discovery of potential therapeutic targets for STSS associated with *S. suis*.

## Introduction

*Streptococcus suis* has been recognized as an emerging zoonotic pathogen (Staats et al., 1997; Lun et al., 2007; Segura, 2009; Wertheim et al., 2009; Gottschalk et al., 2010). Since the first human infection was identified in 1968 in Denmark (Perch et al., 1968), more than 400 cases of *S. suis* infection have been reported worldwide during the subsequent four decades (Lun et al., 2007). Most of these cases presented as meningitis, septicemia, endocarditis, arthritis, or pneumonia (Staats et al., 1997; Lun et al., 2007). Two large outbreaks of *S. suis* serotype 2 (*S. suis* 2) infection in 1998 and 2005 in China affected more than 200 people and led to 52 deaths. Most of these deaths were caused by streptococcal toxic shock syndrome (STSS; Hu et al., 2000; Tang et al., 2006; Yu et al., 2006).

Streptococcal toxic shock syndrome associated with group A streptococci (GAS) is well recognized. A massive over-stimulation of T-cells is believed to be associated with STSS (Brown, 2004; Low, 2013). Superantigens (SAgs), including the streptococcal pyrogenic exotoxin serotypes A, C, and G–M as well as the streptococcal mitogenic exotoxin Z are involved in the molecular and pathological mechanism underlying STSS associated with GAS (Brosnahan and Schlievert, 2011). In addition to the SAgs, M protein, which is a highly conserved cell-surface protein of GAS that can induce strong inflammation, might also contribute to the development of STSS (Pahlman et al., 2006). However, *S. suis* is a non-GAS pathogen, and does not contain DNA sequences that are homologous to the genes encoding the SAgs or M protein, indicating that molecules other than the SAgs or M protein might be involved in the mechanism underlying the STSS outbreaks associated with *S. suis* in China (Tang et al., 2006).

Streptococcal toxic shock syndrome caused by *S. suis* 2, is characterized by acute high fever, vascular collapse, hypotension, shock, petechia, disseminated intravascular coagulation, and multiple organ failure (Tang et al., 2006; Yu et al., 2006). Vascular leakage is a fundamental mechanism of shock. Active polymorphonuclear neutrophils (PMNs) have been shown to release a broad spectrum of cytokines and other molecules that induce increased vascular permeability (Gautam et al., 1998, 2000, 2001). One of these molecules, heparin-binding protein (HBP), is thought to be a key mediator that induces vascular leakage (Gautam et al., 2001; Edens and Parkos, 2003). HBP is also known as azurocidin or CAP37, and has diverse functions. It is usually stored in the azurophilic granules and secretory vesicles in the PMNs (Tapper et al., 2002). The molecular mechanism underlying the release of HBP from the PMNs has been extensively investigated. Bacterial-derived M protein has been shown to bind to fibrinogen and interact with the ß_2_-integrins on the surface of the PMNs, stimulating the release of HBP (Herwald et al., 2004). HBP can also be released from the PMNs by other mechanisms, including PMN degranulation mediated by streptolysin O (Nilsson et al., 2006) and the lipid leukotriene B4 (LTB4)-mediated stimulation of the BLT1 receptor and phosphatidylinositol 3-kinase (PI3K) intracellular pathway (Di Gennaro et al., 2009). The molecular and pathological mechanisms underlying STSS that are not associated with GAS remain poorly understood (Hashikawa et al., 2004; Ekelund et al., 2005). In this study, we focused on the highly virulent *S. suis* 2 strain 05ZYH33 to investigate the molecular mechanism underlying the release of HBP from the PMNs and the induced vascular leakage. This strain was originally isolated from a patient who died from STSS during the *S. suis* outbreak in 2005 in Sichuan, China.

## Materials and Methods

### Reagents

Recombinant human HBP (rHBP, Cat.No.2200-SE-050/CF), polyclonal goat anti-human HBP antibody (Cat.No.AF2200), and monoclonal mouse anti-human HBP antibody (Cat.No.MAB2200) were purchased from R&D (Minneapolis, MN, USA). CLI-095 [a specific inhibitor of Toll-like receptor 4 (TLR4), Cat.No.tlrl-cli95] and OxPAPC (a TLR4 non-specific inhibitor, Cat.No.tlrl-oxp1) were obtained from Invivogen (Hong Kong, China). *Escherichia coli* O26:B6 lipopolysaccharide (LPS, Cat.No.L2654), EGTA (a calcium chelator, Cat.No.E3889), U73122 (a phospholipase C inhibitor, Cat.No.U6756), PD98059 (a mitogen-activated protein kinase pathway inhibitor, Cat.No.P215), SB202474 (a negative control for SB203580), SB203580 (a p38 MAPK inhibitor), Wortmannin (a PI3K inhibitor, Cat.No.F9128) and Genistein (a receptor tyrosine kinase inhibitor, Cat.No.G6649) were purchased from Sigma–Aldrich (St. Louis, MO, USA). Pertussis toxin (PTX; a receptor inhibitor coupled to G protein) was kindly provided by the National Vaccine and Serum Institute (Beijing, China). The PMN Elastase Human ELISA Kit (Cat.No.ab119553) and the Lactoferrin Human ELISA kit (Cat.No.ab108882) were from Cayman Chemical (Ann Arbor, MI, USA).

### Blood Specimens from Patients

All the blood specimens were kindly provided by the Chinese Center for Disease Control and Prevention and kept anonymous. The Chinese Center for Disease Control Human Research Protection Office approved the retrospective testing using anonymous samples. Blood specimens from 13 healthy individuals and 14 patients, including eight with meningitis and six with STSS, were used in this study.

### Isolation of Polymorphonuclear Neutrophils (PMNs)

Human PMNs were isolated from freshly heparinized blood that was collected by gradient centrifugation. Red blood cells were separated by 6% dextran (Sigma–Aldrich), and then the PMNs were isolated using density gradient media containing 70% Percoll (Pharmacia, New York, NY, USA) and Ficoll (GE Healthcare, Little Chalfont, UK). The purified PMNs were suspended in Hanks’ balanced salt solution (HBSS, Invitrogen, Carlsbad, CA, USA) at a concentration of 2 × 10^6^ cells/mL. The cellular purity was greater than 97% as indicated by Wright–Giemsa staining. The survival rate was greater than 97% as assessed by Trypan blue staining. The purified PMNs were used in the following experiments.

### PMN Stimulation

One hundred microliters of human whole blood or 1.0 × 10^6^ isolated PMNs were diluted in HBSS to a final volume of 1.0 mL and incubated with various putative stimulants at 37°C for 30 min. The HBP levels in the blood samples or the release of HBP from PMNs were subsequently determined. Cells were centrifuged at 300 × *g* for 15 min and HBP levels in the supernatant were analyzed by a sandwich ELISA or a Western blot. The amount of HBP in the cell lysate of the whole blood samples or the PMNs obtained by treatment with 1% Triton X-100 was determined and considered as the total HBP. The percentage of the HBP level in the supernatant relative to the total HBP level was calculated.

### Determination of HBP Levels by ELISA

The HBP levels in the supernatant of the whole blood sample or the PMN suspension were determined by a sandwich-based ELISA as previously described (Tapper et al., 2002). Briefly, microtiter plates (Costar, Corning Inc., Corning, NY, USA) were coated with a polyclonal goat anti-human HBP antibody (100 μL of 0.5 μg/mL antibody solution). After washing and blocking, 100 μL of sample was added to duplicate wells and incubated at 37°C for 1 h or 4°C overnight. The standard for quantifying the HBP levels was rHBP. After a thorough washing, the plates were incubated with a monoclonal mouse anti-human HBP antibody (100 μL of 1 μg/mL antibody solution) at 37°C for 1 h or 4°C overnight. A horseradish peroxidase (HRP)-conjugated antibody against mouse IgG (1:8,000, ZSGB-Bio, Beijing, China) was added to detect the primary antibody. The plate was read in a microtiter plate reader (Thermo Fisher Scientific, Waltham, MA, USA) at 450 nm.

### Detection of HBP by Western Blotting

Thirty microliters of the supernatant of a whole blood sample or a PMN suspension were separated by 12% (w/v) polyacrylamide gel electrophoresis in the presence of 1% (w/v) sodium dodecyl sulfate (SDS-PAGE) and transferred to a polyscreen polyvinylidene difluoride membrane (Merck Millipore, Billerica, MA, USA). The membrane was first blocked in 5% (w/v) non-fat milk in phosphate-buffered saline (PBS) supplemented with 0.05% (v/v) Tween 20 at room temperature for 1 h, and incubated with the polyclonal goat anti-human HBP antibody (1 μg/mL) at 4°C overnight. After intensive washing, the membrane was incubated with HRP-labeled mouse anti-goat IgG (1:8,000, ZSGB-Bio) for 1 h. The immunoblots were analyzed using a Gel Imaging System and the Quantity One Software version 4.0 (Bio-Rad, Hercules, CA, USA).

### Bacterial Strains and Culture Conditions

The highly virulent *S. suis* serotype type 2 strain 05ZYH33 (GenBank accession number NC_009442), which was originally isolated from a patient who died from STSS during the *S. suis* outbreak in 2005 in Sichuan, China, was used in this study. A suilysin gene (*sly*) deletion mutant of 05ZYH33 (Δ*sly*) was constructed via the in-frame replacement of the SSU05_1403 gene with a chloramphenicol (Cm) resistance cassette. The complement strain of the Δ*sly* mutant (CΔ*sly*) was established by transforming the mutant with a pAT18 vector that expressed the *sly* gene. These strains were previously constructed in our laboratory (He et al., 2014). All the bacterial strains were grown on Colombia agar plates (BD Biosciences, San Jose, CA, USA) containing 5% sheep’s blood at 37°C under 5% CO_2_. The bacterial suspensions were grown in Todd-Hewitt broth (THB, BD) for 8 h without agitation, and the supernatants were harvested for future experiments. Five microgram per mL of Cm or 8 μg/mL Em (Sigma–Aldrich) were added to the media for selection.

### Purification of Native and Recombinant Suilysin (SLY)

Native SLY was purified as previously described (Jacobs et al., 1994; Lv et al., 2014). Briefly, the supernatant from a large-scale 05ZYH33 culture was collected by continuous-flow centrifugation and filtered through a ceramic filer with a 0.8 μm pore size at 4°C, and concentrated to 150 ml using 10,000-nominal-molecular-weight-limit filters (PTCG; Minitan; Merck Millipore). The concentrated supernatants were sterilized by filtration through a 0.2 μm pore size filer (Falcon, Thermo Fisher Scientific), loaded onto a Superose-12 gel filtration column (FPLC, Pharmacia), and eluted into 40 mM PBS (pH 7.2) supplemented with 0.5 M NaCl. The elution fractions were collected and analyzed by SDS-PAGE. The recombinant SLY and the non-hemolytic *sly* mutant SLY (P353V) used in this study were previously constructed and purified in our laboratory (Ren et al., 2012). The endotoxin level in the native suilysin (SLY) was less than 0.03 EU/mL. The endotoxins that remained in the purified recombinant SLY were removed with Triton X-114 (Liu et al., 1997) and were less than 0.5 EU/mL before the purified recombinant SLY was used in experiments.

### Measurement of Superoxide Anion Production

Superoxide anion production was determined as previously described (Nilsson et al., 2006). PMNs were stimulated with 0.50, 0.75, or 1.0 μg/mL native SLY or with the supernatants from 05ZYH33 cultures at 37°C for 20 min. The PMNs were kept on ice for 10 min to terminate the stimulation, collected by centrifugation at 200 × *g* for 15 min and re-suspended in 0.25 ml Krebs-Ringer phosphate (KRG, 120 mM NaCl, 4.9 mM KCl, 1.7 mM KH_2_PO_4_, 1.2 mM MgSO_4_⋅7H_2_O, 8.3 mM Na_2_HPO_4_⋅2H_2_O, 10 mM glucose, pH 7.3) buffer containing 0.1 mM cytochrome C. Superoxide production from the PMNs was induced by incubation with 200 ng/mL phorbol myristate acetate (PMA) at 37°C for 20 min. The PMNs were pelleted, and the absorbance of the supernatants was determined at 550 nm. To determine baseline levels of superoxide anion production, the PMNs were incubated with KRG buffer without cytochrome C. KRG buffer without PMNs was used as the blank reference. The HBP in the supernatant was also determined by Western blotting.

### Electron Microscopy

The integrity of the PMNs was assessed by electron microscopy. After incubation with 1.0 μg/mL native SLY, the PMNs were spread onto poly-L-lysine coated coverslips, incubated at 37°C for 1 h, and fixed in 2.5% (v/v) glutaraldehyde at room temperature for 30 min. The PMNs were dehydrated in an ascending ethanol series from 50% (v/v) to absolute ethanol (10 min per step). The specimens were subjected to critical point drying in CO_2_ with absolute ethanol as an intermediate solvent, mounted on copper holders, sputtered with 30 nm palladium and gold, and examined in an S-3400N scanning electron microscope.

### Flow Cytometry

A total of 2 × 10^6^ PMNs were incubated with 1.0 μg/mL native SLY at 37°C for 30 min with rotation and subsequently kept on ice. The expression of markers for PMN degranulation was assessed with PE conjugated mouse anti-human CD63 (1:100), APC conjugated mouse anti-human CD11b (1:100), and FITC conjugated mouse anti-human CD66b (1:100, BD Biosciences). Isotype-matched antibodies were used as negative controls. The fluorescence intensity of each sample was determined on an Accuri C6 flow cytometer (BD Biosciences). The data were analyzed using the FlowJo software (FlowJo LLC, Ashland, OR, USA).

### Determination of Calcium Mobilization

Polymorphonuclear neutrophils were loaded with 5 μM Fura-3 AM (Invitrogen) in HBSS at 37°C for 1 h and plated into a confocal dish (Thermo Fisher Scientific). Putative PMN stimulants were added to the confocal dish. The PMNs were monitored with a FV1000 confocal laser-scanning microscope (Olympus, Tokyo, Japan) for 200 s. The mean fluorescence intensity of at least eight cells was recorded.

### *In vivo* Miles Assay for the Assessment of Vascular Permeability

The Miles assay was performed on 8-week-old female C57BL/6J mice to evaluate the effects of SLY on vascular permeability. C57BL/6J mice were purchased from the Animal Care Center of the Academy of Military Medical Science (AMMS; Beijing, China) and housed in a clean room with unlimited access to food and water. TLR4 knockout mice C57BL/10ScNJNju and control mice were obtained from the Model Animal Research Center of Nanjing University. The mice were randomly divided into four groups and intradermally injected in the abdomen with 100 μL THB, 05ZYH33-supernatant, Δ*sly*-supernatant, or CΔ*sly*-supernatant. Four hours after the injection, 100 μL Evans blue dye solution (2.5%, Ourchem, Sinopharm) was injected via the tail vein. The mice were sacrificed by cervical dislocation 30 min after the Evans blue injection, and equal areas (20 mm × 20 mm) of skin surrounding the intradermal injection site were removed from each mouse and completely dried in an oven. The Evans blue dye was eluted from the oven-dried skin into 1 mL formamide (Sinopharm) at 55°C for 2 days, and quantified by spectrophotometry at 630 nm.

### Statistics

Statistical analyses were performed using the Prism 5 software (GraphPad Software, La Jolla, CA, USA). For normally distributed data, a comparison between two groups was made using Student’s *t*-test, and comparisons among multiple groups were done with a one-way analysis of variance. The *P*-value was for a 2-sided test, and *P* < 0.05 was considered as statistically significant. All data were expressed as the mean ± standard deviation. A rank-sum test was performed to compare the HBP levels in the patients versus the controls.

### Study Approval

All the protocols for handling the patient blood specimens or experiments using blood from healthy donors were approved by the Institutional Medical Ethics Committee of AMMS. A signed informed consent form was obtained from each patient or a guardian and healthy volunteer. The entire study was conducted in accordance with the Declaration of Helsinki.

All experimental procedures involving mice were conducted in strict accordance with the recommendations in the Guide for the Care and Use of Laboratory Animals of the National Institutes of Health and State Key Laboratory of Pathogens and Biosecurity of the Beijing Institute of Microbiology and Epidemiology and approved by the Institutional Animal Care and Use Committee of the AMMS under Permit No. IACUC of AMMS-2014-031. Animal welfare was considered and suffering was minimized.

## Results

### Suilysin from the Strain 05ZYH33 Induced the Release of HBP from PMNs

**FIGURE 1:**
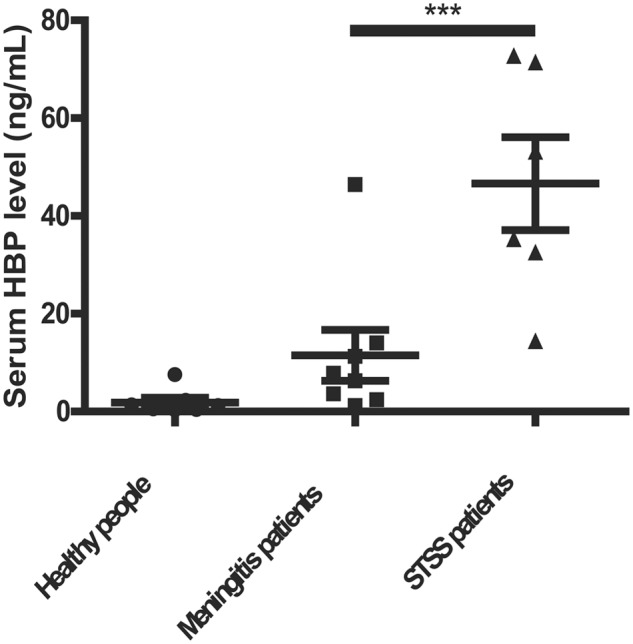
**The serum HBP levels were significantly increased in patients with STSS compared with patients without STSS or healthy individuals.** The serum HBP levels of healthy individuals (*n* = 13), patients infected with *S. suis* presenting meningitis (*n* = 8), and patients infected with *S. suis* presenting STSS (*n* = 6) were determined by ELISA. Data were analyzed by a rank-sum test. ^∗∗∗^ indicates a significant difference between patients with STSS vs. controls, *P* = 0.0007.

**FIGURE 2:**
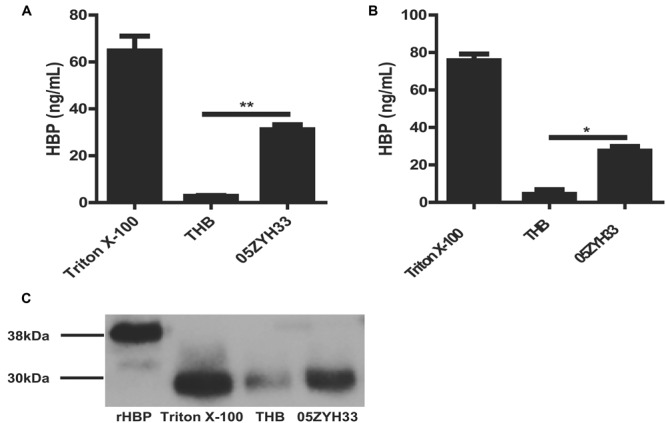
**05ZYH33 cultures induced the release of HBP in whole blood samples and purified PMN suspension. (A,B)** 05ZYH33 cultures significantly induced the release of HBP in human whole blood samples **(A)** and purified PMN suspensions **(B)**. Whole blood samples or purified PMN suspensions were incubated with 05ZYH33 cultures or THB (control) for 30 min at 37°C. The HBP level in the supernatants was measured by ELISA. The total cell lysate following Triton X-100 treatment was used as the positive control. Data are expressed as the mean ± SD. An unpaired Student’s *t*-test was used. ^∗∗^*P* < 0.01, ^∗^*P* < 0.05. **(C)** Western blotting results of release of HBP from human purified PMN suspensions. rHBP and Triton X-100 were used as positive controls.

Heparin-binding protein has been reported as a potential biomarker of severe sepsis and septic shock (Linder et al., 2009, 2012; Chew et al., 2012) and as an indicator of vascular leakage (Johansson et al., 2009). A comparison of serum HBP levels between patients with *S. suis*-associated STSS versus patients without STSS or healthy individuals revealed that the serum HBP levels in patients with STSS were significantly higher than those in patients with meningitis or healthy controls (46.61 ± 9.49 ng/mL vs. 11.52 ± 5.20 ng/mL or 1.84 ± 0.96 ng/mL, respectively; *P* = 0.0007, **Figure 1**), which suggested that HBP is released during the progression of STSS in patients infected with *S. suis*. Both the ELISA and Western blotting analyses demonstrated that the bacterial culture of the highly virulent *S. suis* strain 05ZYH33 significantly increased the release of HBP in the whole blood of healthy individuals (**Figure 2A**) and in purified PMN suspension (**Figures 2B,C**), whereas THB media without 05ZYH33 failed to induce the release of HBP in a whole blood sample or a PMN suspension. The positive control for the Western blot was rHBP. The molecular weight of native HBP is approximately 31 kDa, while rHBP appears to be approximately 39 kDa on a Western blot owning to its carboxyl-terminal polyhistidine tag (**Figure 2C**). These results suggest that strain 05ZYH33 stimulates the release of HBP from PMNs.

**FIGURE 3:**
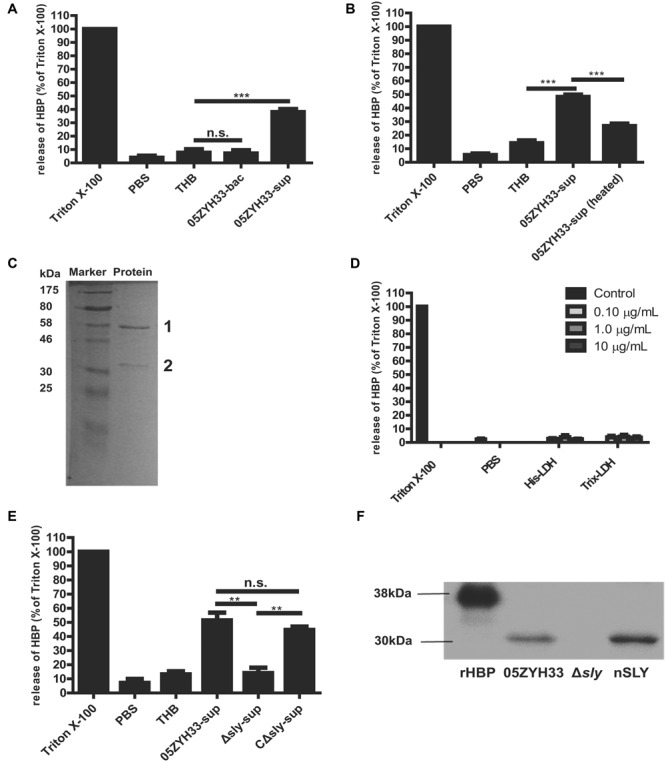
**Suilysin secreted from 05ZYH33 induced the release of HBP from PMNs. (A)** The release of HBP from whole blood samples was induced by supernatants of 05ZYH33 but not by the bacterial cells. Human whole blood samples were incubated with PBS, THB, 05ZYH33 bacterial cells, or 05ZYH33 supernatants for 30 min at 37°C. The HBP level in the supernatants was measured by ELISA. PBS was considered as the background and THB was used as a negative control. An unpaired Student’s *t*-test was used for the statistical analysis. **(B)** Heat treatment of the supernatants significantly reduced the release of HBP from whole blood samples. The supernatants were treated at 100°C for 5 min before being added to whole blood samples. **(C)** A SDS-PAGE analysis of the elution fraction from anion-exchange and hydrophobic interaction chromatography, which induced the maximal release of HBP. **(D)** LDH failed to induce the release of HBP from PMNs. The PMNs were incubated with His-LDH or Trix-LDH at 0.1, 1.0, or 10 μg/mL for 30 min at 37°C. **(E)** Suilysin in the 05ZYH33-supernatant stimulated the PMNs to secret HBP. Human whole blood samples were incubated with PBS, THB, the 05ZYH33-supernatant, the Δ*sly*-supernatant or the CΔ*sly*-supernatant for 30 min at 37°C. **(F)** Western-blot analysis of the HBP that was released from PMNs incubated with cultures of wild-type 05ZYH33, the Δ*sly* mutant, or purified nSLY. A representative image is presented. ^∗∗∗^*P* < 0.001, ^∗∗^*P* < 0.01, n.s., not significant. ELISA data are expressed as the mean ± SD of at least four independent experiments.

The molecule responsible for inducing the release of HBP was identified. Supernatants of the 05ZYH33 culture, but not the 05ZYH33 bacterial cells, significantly induced the release of HBP in whole blood samples (*P* < 0.001, **Figure 3A**). Heat treatment of the supernatants significantly reduced the release of HBP in the whole blood samples (*P* < 0.001, **Figure 3B**), indicating that the specific factor(s) could be proteins. Therefore, the proteins in the supernatants were isolated by anion-exchange and hydrophobic interaction chromatography. The proteins in the elution fractions that markedly stimulated the release of HBP were separated by SDS-PAGE, and two major protein bands at approximately 58 and 35 kDa appeared on the gel (**Figure 3C**). Mass spectrometry revealed that the 58 kDa protein was SLY, and the 35 KDa protein was L-lactate dehydrogenase (LDH; Supplementary Table S1). Recombinant LDH failed to stimulate the release of HBP from PMNs (**Figure 3D**), which suggests that LDH was not the molecule that induced the release of HBP. Therefore, SLY is a likely candidate for the substance that releases HBP. Supernatants of the *sly* gene deletion mutant strain of 05ZYH33 (Δ*sly*) did not stimulate the release of HBP, while the supernatants of the complement strain for Δ*sly* (CΔ*sly*) restored the stimulation of the release of HBP (*P* < 0.01, **Figure 3E**). Furthermore, Western blotting showed that purified native SLY (nSLY) stimulated the release of HBP from the PMNs, but a bacterial culture of Δ*sly* strain failed to do so (**Figure 3F**). Taken together, these findings suggest that SLY secreted by 05ZYH33 induces the release of HBP from the PMNs.

### Suilysin Stimulated the Release of HBP by Promoting PMN Degranulation

**FIGURE 4:**
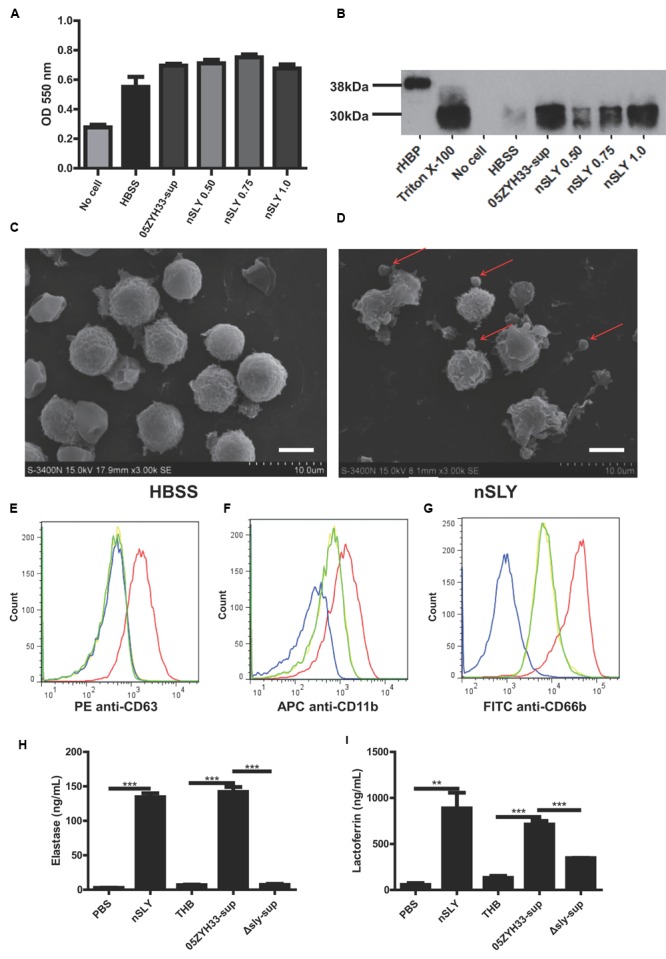
**Suilysin induced PMN degranulation. (A)** Superoxide anion production was not affected by treatment with nSLY or 05ZYH33 supernatants. Purified PMNs were incubated with 0.50, 0.75, or 1.0 μg/mL nSLY, the 05ZYH33-supernatant, or HBSS for 30 min at 37°C. Following treatment, the cells were stimulated with 200 ng/ml PMA in the presence of 0.1 mM cytochrome C. The superoxide anion production was determined by measuring the absorbance at 550 nm. Data are expressed as the mean ± SD of three independent experiments. **(B)** Western blot analysis of release of HBP from PMNs that were treated as described for the measurement of superoxide anion production. Thirty microliters of each sample was loaded in each lane. A representative image is presented. **(C,D)** Scanning electron microscopy images of purified PMNs that were pre-incubated with HBSS **(C)** or 1 μg/mL nSLY **(D)**. Red arrows indicate excessive membrane ruﬄes. Representative images are presented. The scale bar represents 5 μm. **(E–G)**. Levels of membrane-associated marker proteins for PMN degranulation, CD63 **(E)**, CD11b **(F)**, and CD66b **(G)** were increased by 1 μg/mL rSLY. PMNs were stimulated with HBSS as a negative control (—), SLY-isotype (—), rSLY (—), or rFhb (—) and analyzed by flow cytometry. **(H,I)** Levels of granule marker proteins for PMN degranulation, elastase **(H)** and lactoferrin **(I)** were increased by 1 μg/mL nSLY or the 05ZYH33 supernatant. ^∗∗∗^*P* < 0.001, ^∗∗^*P* < 0.01. ELISA data are presented as the mean ± SD of at least three independent experiments.

Heparin-binding protein is usually stored in azurophilic granules and secretory vesicles in the PMNs and released by cell lysis or via degranulation after the activation of a series of signal transduction pathways (Borregaard and Cowland, 1997; Tapper et al., 2002). We can infer that a high concentration of SLY could lyse the PMNs and release the stored HBP, while a sub-cytolytic concentration would interact with the cell membrane receptor and could possibly trigger PMN degranulation. Different concentrations of nSLY (0.50–1.0 μg/mL) did not affect the production of superoxide anions from the PMNs (**Figure 4A**), while the release of HBP from the PMNs was SLY dose-dependent (**Figure 4B**), suggesting that the PMNs remained viable when treated with nSLY. These findings indicate that cell lysis is not related to the release of HBP mediated by SLY. Scanning electron microscopy demonstrated that the PMNs exhibited membrane blebs when treated with 1.0 μg/mL nSLY (**Figure 4D**) and the control did not exhibit such blebs (**Figure 4C**), suggesting that the degranulation might be induced by nSLY. A flow cytometry analysis revealed the significant up-regulation of markers for azurophilic granules (CD63), secondary/tertiary granules and secretory vesicles (CD11b), and specific granules (CD66b, also named CD67) at the plasma membrane after the PMNs were incubated with recombinant SLY (rSLY), but the recombinant factor H-binding protein (rFhb – a *S. suis* cell wall protein that was purified using the same methods) was not up-regulated. This implies that the granular vesicles in the PMNs were mobilized for degranulation (**Figures 4E–G**). Other proteins stored in the neutrophil granules, including elastase from the azurophilic granules and lactoferrin from the secondary granules, were released when the neutrophils were incubated with nSLY or the 05ZYH33-supernatant (**Figures 4H,I**). All of these results suggest that SLY induces the release of HBP via PMN degranulation.

### The Release of HBP Induced by Suilysin Was Dependent on the Ca^2+^ Influx

**FIGURE 5:**
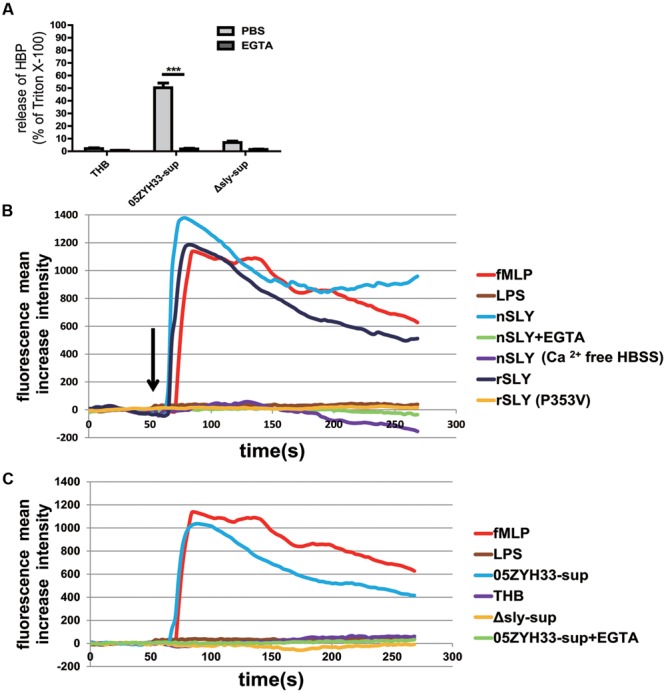
**SLY induced a Ca^2+^ influx and the release of HBP was dependent on the Ca^2+^ influx. (A)** The SLY-induced release of HBP from human whole blood samples was abolished by EGTA. PMNs were pre-incubated with EGTA (10 mM) for 1 h at 37°C. ^∗∗∗^*P* < 0.001. **(B)** SLY induced a Ca^2+^ influx. Positive control fMLP, negative control LPS, nSLY, nSLY with pre-incubation of EGTA or with Ca^2+^-free HBSS, rSLY, non-hemolytic SLY mutant P353V was added to a confocal small dish containing purified PMNs loaded with the fluorescent Ca^2+^ indicator Fluo-3/AM at the indicated time (60 s, arrow), and Ca^2+^ mobilization was monitored by real-time fluorescence microscopy for 250 s. **(C)** SLY in the 05ZYH33-supernatant stimulated a Ca^2+^ influx. The positive control was fMLP, the negative control was LPS, and the 05ZYH33-supernatant, THB, the Δ*sly*-supernatant, the 05ZYH33-supernatant with pre-incubation of EGTA were used as stimulants. The fluorescence intensity of at least eight cells in one vision was measured and the mean increase intensity is presented.

An increased cytosolic Ca^2+^ concentration has been shown to promote PMN degranulation via the mobilization of intracellular granules and cytoskeleton rearrangements (O’Flaherty et al., 1991; Lacy, 2006; Lacy and Eitzen, 2008). Thus, the SLY-induced release of HBP might also be Ca^2+^-dependent. Indeed, the release of HBP induced by the bacterial culture supernatants was completely abolished in the presence of EGTA (*P* < 0.0001, **Figure 5A**), suggesting that the release of HBP is dependent on extracellular Ca^2+^. The measurement of intracellular Ca^2+^ revealed that nSLY (1 μg/mL) induced a rapid increase in the intracellular Ca^2+^ concentration in standard HBSS, and that the Ca^2+^ increase was completely abolished by EGTA or in Ca^2+^-free HBSS (**Figure 5B**). *N*-formylmethionyl-leucylphenylalanine (fMLP) served as a positive control and LPS was used as a negative control (**Figures 5B,C**). These results indicate that SLY induced the Ca^2+^ influx. A constructed non-hemolytic *sly* mutant SLY (P353V; Xu et al., 2010; Du et al., 2013) could neither induce calcium mobilization (**Figure 5B**), nor evoke the secretion of HBP (Supplementary Figure S1), suggesting that the SLY-induced release of HBP was dependent on the Ca^2+^ influx. Supernatants of 05ZYH33 consistently induced a Ca^2+^ influx, whereas THB media and supernatants of the *Δsly* mutant did not induce a Ca^2+^ influx (**Figure 5C**). The Ca^2+^ influx induced by the 05ZYH33 supernatant was also completely abolished by EGTA (**Figure 5C**). All of these results indicated that the Ca^2+^ influx plays a critical role in the release of HBP induced by SLY.

### The Release of HBP Induced by Suilysin Was Dependent on the TLR4 Receptor, p38 MAPK, and the PI3K Pathways, as well as GPCR

To investigate possible mechanisms and the key molecules involved in release of HBP, inhibitors were commonly used in this study because of the lack of suitable cell lines for investigating the release of HBP induced by SLY, and direct siRNA transfection into PMNs could cause activation of the PMNs and the release of HBP (data not shown).

**FIGURE 6:**
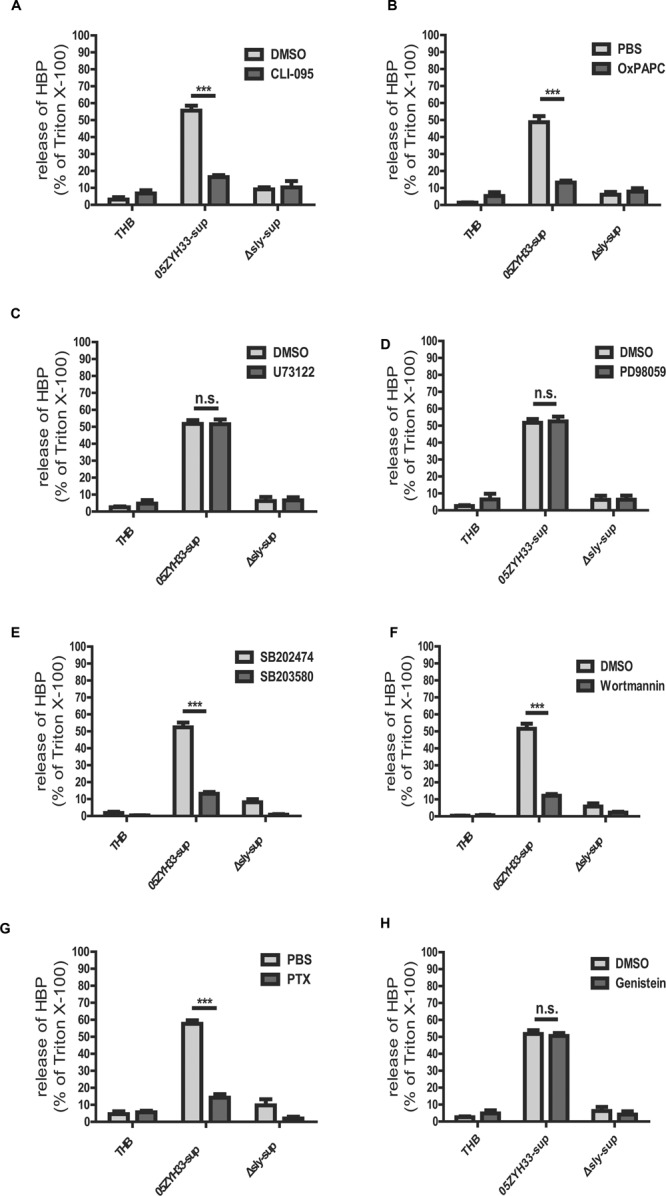
**Receptor and signal molecule were involved in the Suilysin induced release of HBP. (A,B)** The release of HBP was blocked by the TLR4-specific inhibitor CLI-095 (3 μM; **A**) and the TLR4-non-specific inhibitor OxPAPC (30 μg/mL; **B**). **(C,D)** The release of HBP was not affected by the PLC and PLA2 inhibitor U73122 (10 μM; **C**) and the ERK1/2 inhibitor PD98095 (20 μM; **D**). (**E–H)** The release of HBP was blocked by the p38 MAPK inhibitor SB203580 (10 μM; **E**), the PI3K inhibitor Wortmannin (1.0 μM; **F**), the GPRP inhibitor PTX (0.15 μg/mL; **G**) but not by the RTK inhibitor Genistein (100 μM; **H**). The PMNs were pre-treated with or without the inhibitors at 37°C for 1 h (PTX for 3h and CLI-095 for 6 h) before a 30 min incubation with the stimulants at 37°C. The HBP level was measured by ELISA. The HBP level relative to the total amount of HBP in the cell lysate from treatment with Triton X-100 was calculated. The data are expressed as the mean ± SD of at least four independent experiments. ^∗∗∗^*P* < 0.001. n.s., not significant.

Some cholesterol-dependent cytolysin (CDC) family members, such as anthrolysin O, perfringolysin O, listeriolysin O, streptolysin O, and pneumolysin, have been reported to interact with TLR4 (Malley et al., 2003; Park et al., 2004; Srivastava et al., 2005). SLY also belongs to the CDC family, and results from our previous study have shown that SLY interacts with TLR4 to induce an inflammatory response (Bi et al., 2015). In this study, our results demonstrated that both the TLR4 specific inhibitor CLI-095 (**Figure 6A**), which blocks the intracellular domain of TLR4, and the TLR4 non-specific inhibitor OxPAPC (**Figure 6B**), which competes with other ligands including CD14, LBP, and MD2 for receptor binding, significantly reduced the release of HBP induced by the 05ZYH33 supernatant. The results for both inhibitors were significant at *P* < 0.0001. This suggests that the release of HBP induced by SLY could be dependent on TLR4. We then used a pharmacological approach to further investigate the signaling pathway underlying the SLY-induced release of HBP. The phospholipase C inhibitor U73122 (**Figure 6C**) and the MAPK pathway inhibitor PD98059 (**Figure 6D**) did not affect the release of HBP, whereas the p38 MAPK inhibitor SB203580 (**Figure 6E**) and the PI3K inhibitor Wortmannin (**Figure 6F**) markedly reduced HBP secretion (both *P* < 0.0001). We confirmed that a time-dependent increase in the level of phosphorylated p38 MAPK occurred in the presence of nSLY (1.0 μg/ml; Supplementary Figure S2). Other receptor inhibitors such as the GPCR inhibitor PTX significantly inhibited the release of HBP (*P* < 0.0001, **Figure 6G**), but Genistein, an inhibitor of the receptor tyrosine kinases (RTK), had no effect (**Figure 6H**). Therefore, a GPCR might also be involved in the release of HBP induced by SLY in addition to TLR4. The mechanism could be complex, and further studies are necessary to explain why two receptors are involved in the release of HBP induced by SLY.

### Suilysin Increased Vascular Leakage in Mice

**FIGURE 7:**
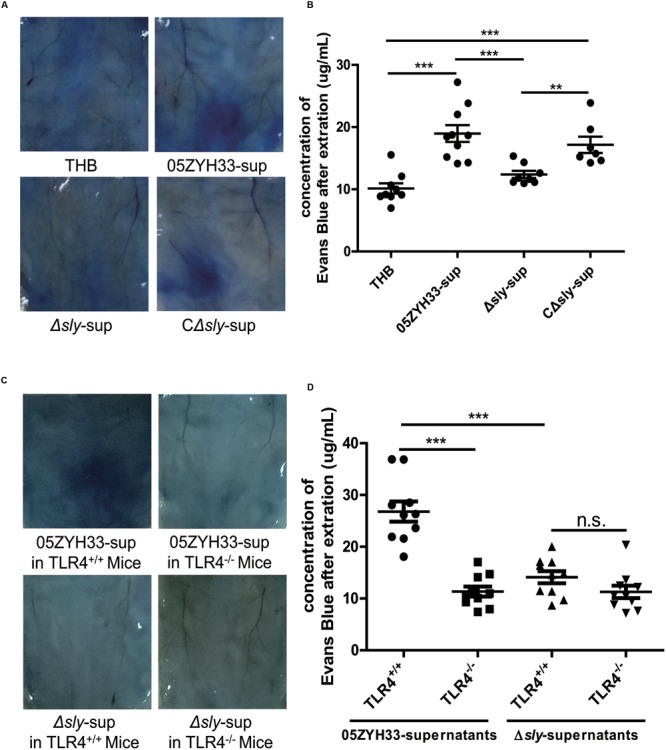
**Suilysin increased the vascular leakage in C57BL mice. (A,B)** SLY increased the vascular leakage in mice. C57BL/6J mice were intradermally injected with THB, the 05ZYH33-supernatant, the Δ*sly*-supernatant, or the CΔ*sly*-supernatant. Four hours later, Evans blue dye (2.5%, 100 μL) was injected via the tail vein. After 30 min, photographs of the skin area containing the extravasated protein-bound dye were taken, and the dye was extracted from the skin using formamide. Dye concentrations were measured at 630 nm using a spectrophotometer. **(C,D)** The 05ZYH33 supernatant did not induce vascular leakage in *Tlr4* knockout mice. TLR4^+/+^ and TLR4^-/-^ were treated in the similar ways as in **(A,B)**. ^∗∗∗^*P* < 0.001, ^∗∗^*P* < 0.01, n.s., not significant.

The effects of SLY on vascular leakage were tested in 8-week-old female C57BL mice using the Miles assay. Supernatants of the wild-type 05ZYH33 strain and the C*Δsly* mutant significantly increased the leakage of Evans blue dye in mice compared with a THB control or the supernatants from the Δ*sly* mutant (05ZYH33-sup: 18.96 ± 1.35 μg/mL vs. Δ*sly*-sup: 12.38 ± 0.57 μg/mL, *P* = 0.0008, **Figures 7A,B**), implying that SLY contributes to vascular leakage. In addition, the 05ZYH33 supernatant failed to induce vascular leakage in *Tlr4* knockout mice (TLR4^+/+^: 26.80 ± 1.96 μg/mL vs. TLR4^-/-^: 11.35 ± 0.98 μg/mL, *P* < 0.0001, **Figures 7C,D**). These *in vivo* data support the essential role of TLR4 in vascular leakage mediated by SLY.

## Discussion

Since the two large outbreaks of *S. suis* human infections occurred in China, sporadic cases of *S. suis*-associated STSS have been reported worldwide (Tramontana et al., 2008; Gomez et al., 2014). This indicates that *S. suis* has already become a persistent problem, and the potential for a large-scale outbreak exists. Although a great deal of effort has been spent on *S. suis*, the pathogenic mechanism of STSS associated with *S. suis* remains poorly understood. The results of this study demonstrated that SLY secreted by the highly virulent *S. suis* strain 05ZYH33 stimulated the release of HBP from the PMNs and induced vascular leakage in mice, indicating a role for SLY in the development of STSS. An in-depth investigation of the molecular mechanism underlying the release of HBP from PMNs mediated by SLY revealed that the release of the HBP occurred via Ca^2+^-influx dependent degranulation and required both the p38 MAPK and PI3K signaling pathways.

Suilysin is a member of the CDC toxin family. It comprises 497 amino acids and exhibits hemolytic activity. SLY was considered as a putative virulence factor when it was first purified (Jacobs et al., 1994). However, studies investigating the association of SLY with the virulence of *S. suis* yielded conflicting results. A *sly* deletion mutation did not appear to affect the virulence of *S. suis* in piglets although it did impact the virulence in mice (Allen et al., 2001; Lun et al., 2003). Immunization with purified SLY failed to induce significant protection against *S. suis* infection in piglets (Jacobs et al., 1996). Moreover, virulent *S. suis* strains from North America do not have the *sly* gene in their genome (Gottschalk et al., 1998). These findings seem to support the view that SLY is not an essential virulence factor of *S. suis*. In contrast, we conducted a study in which SLY levels in different *S. suis* strains were compared. That study showed that the highly virulent strain 05ZYH33, which caused higher mortality and greater damage to PMNs and human umbilical vein endothelial cells than non-epidemic strains, expressed a higher level of SLY than less virulent strains (He et al., 2014). In addition, a s*ly* deletion markedly reduced the virulence of 05ZYH33 in mice (He et al., 2014). However, Gottschalk et al. thought that the higher toxicity of the Chinese ST7 strain for human peripheral blood mononuclear cells could simply be the result of its higher capacity to release SLY (Ye et al., 2006, 2009; Gottschalk et al., 2010). These results suggest that SLY is associated with the high virulence of the Chinese strain.

The role of SLY in the mediation of the inflammatory response is also controversial. Segura et al. (2006) suggested that SLY might only play a limited role in stimulating the release of pro-inflammatory cytokines, and the consequent inflammatory response. They found an increase in the mRNA and protein levels of pro-inflammatory cytokines that did not appear to be affected in a SLY-negative mutant strain compared with a wild-type *S. suis* strain (Segura et al., 2006). They also showed that purified SLY did not stimulate the production of tumor necrosis factor alpha (TNF-α) or interleukin-6 (IL-6) in murine macrophages (Segura et al., 1999). In contrast, Lun et al. found that rSLY triggered the release of TNF-α and IL-6 from human and pig monocytes (Lun et al., 2003). The intraperitoneal injection of a recombinant SLY resulted in a significant increase in the serum IL-6 levels in C57BL/6 mice (Du et al., 2009). This discrepancy might be attributable to the various SLY activities obtained with different purification methods or because different *S. suis* strains were used in these studies. HBP has been proven to be an important inflammatory mediator that can recruit and activate monocytes (Pereira et al., 1990; Lee et al., 2003; Soehnlein et al., 2005). To our knowledge, SLY from the highly virulent strain 05ZYH33 was found to induce the release of HBP from PMNs for the first time in this study.

In this study, we also found that, in addition to activating intracellular signaling pathways including the p38 MAPK and PI3K pathways, SLY also induced a Ca^2+^ influx in the PMNs. Mobilization of cytosolic free Ca^2+^ and cytoskeletal rearrangement have been demonstrated as requirements for degranulation in PMNs (O’Flaherty et al., 1991; Lacy, 2006; Lacy and Eitzen, 2008). In this study, removal of the extracellular Ca^2+^ by chelation with EGTA completely abolished the release of HBP, further supporting a critical role for a Ca^2+^ influx in PMN degranulation. Furthermore, we found that a non-hemolytic SLY mutant (P353V) failed to induce a Ca^2+^ influx (**Figure 5B**) and the release of HBP (Supplementary Figure S1), also indicating that the release of HBP is Ca^2+^ dependent. By directly monitoring the intracellular Ca^2+^ concentration, we provided direct evidence for the first time to support that SLY induced a Ca^2+^ influx. The underlying mechanism of the Ca^2+^ influx induced by SLY will be determined in future studies.

Until now, the molecular and pathological mechanisms underlying STSS that is not associated with GAS have been poorly understood. In this study, we found that mitilysin secreted from *Streptococcus mitis* and vaginolysin released from *Streptococcus viridans*, also belong to the CDC toxin family and may share a similar crystal structure with SLY, because they share 49 and 51% sequence identities, respectively, with the primary protein sequence of SLY. This indicates that these two toxins may have a similar function in the host cell. These two opportunistic pathogens may induce the release of HBP and cause vascular leakage when they enter the human bloodstream, and lead to STSS with severe vascular leakage. To some extent, these phenomena may be involved in the STSS cases caused by *S. mitis* in 1991 in the Yangtze River Delta of China (Lu et al., 2003). On the other hand, other CDC toxins such as anthrolysin O, perfringolysin O, listeriolysin O, and streptolysin O, may also lead to the release of HBP in humans. Because HBP has already been considered as a potential biomarker for septic shock (Linder et al., 2009, 2012; Chew et al., 2012), these toxins could be a possible reason for the release of HBP in septic shock caused by related strains.

In summary, we reported for the first time in this study that SLY from the highly virulent *S. suis* strain 05ZYH33 induced the release of HBP from PMNs in a Ca^2+^ influx-dependent manner and increased vascular leakage in mice. We provided new insights into vascular leakage in STSS not associated with the GAS, which might lead to the discovery of potential therapeutic targets for *S. suis*-associated STSS.

## Author Contributions

All authors significantly contributed to this study. SC, WX, KW, PL, HJ, and YJ conceived and designed the entire study. SC, WX, KW, PL, ZR, LL, and YY performed experiments. SC, WX, KW, PL, and CZ analyzed data and prepared Figures. ZR, YZ, and QL provided important reagents. SC wrote the first draft of the paper and YJ revised the manuscript. All authors read and approved the manuscript.

## Conflict of Interest Statement

The authors declare that the research was conducted in the absence of any commercial or financial relationships that could be construed as a potential conflict of interest.
