# Supplementary material for: Suilysin Stimulates the Release of Heparin Binding Protein from Neutrophils and Increases Vascular Permeability in Mice
Source: Front Microbiol. 2016 Aug 26;7:1338. doi: 10.3389/fmicb.2016.01338 (PMC4999480; doi:10.3389/fmicb.2016.01338)
Supplement: Supplementary file 1 [file Data_Sheet_1.PDF]

Spot ID	Accession	Mass	Score	Description	Sequence coverage	matched peptides
1	gi|146319057	54817	183	hemolysin [Streptococcus suis 05ZYH33]	19%	91
2	gi|119359977	35428	440	L-lactate dehydrogenase [Streptococcus suis 05ZYH33]	39%	128

Table S1 The MS identification of the target protein

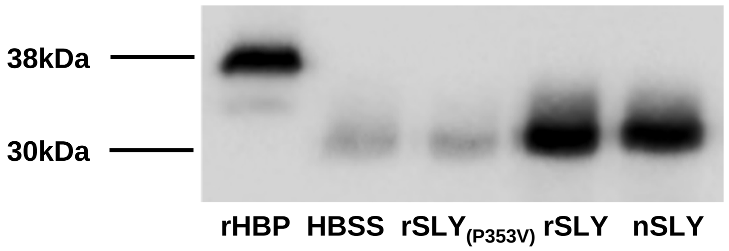

Figure S1 Western-blot analysis of HBP released from PMNs incubated with HBSS, rSLY (P353V), rSLY and nSLY.

The purified PMNs were incubated with HBSS, rSLY (P353V), rSLY, nSLY (1.0 μg/ml) for 30 min at 37° C.

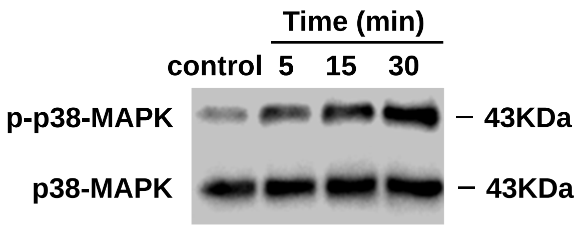

Figure S2 Western-blot analysis of p38 MAPK phosphorylation.

The purified PMNs were incubated with nSLY (1.0 μg/ml) for indicated time at 37° C and the total p38 MAPK and the phosphorylated p38 MAPK were detected.
